# Epidemiology of bacterial meningitis in children admitted to Gondar University Hospital in the post pneumococcal vaccine era

**DOI:** 10.11604/pamj.2018.31.193.10254

**Published:** 2018-11-20

**Authors:** Ashenafi Tazebew Amare, Zemene Tigabu Kebede, Henry Delois Welch

**Affiliations:** 1 Department of Paediatrics, University of Gondar College of Medicine and Health Sciences, Gondar, Ethiopia; 2 Baylor College of Medicine, Houston, USA

**Keywords:** Meningitis, children, resource limited, Ethiopia, pneumococcal vaccine

## Abstract

**Introduction:**

Community acquired bacterial meningitis (CABM) is responsible for high mortality and disabling sequelae. Introduction of pneumococcal conjugate vaccine (PCV-10) and *haemophilus influenzea*type b (Hib) has changed the epidemiological and clinical features of patients presenting with CABM as it is shown in different literatures over the last decade. The aim of this study was to assess the clinical and epidemiologic features and outcomes of CABM after the introduction of PCV-10 in Gondar University Hospital (GUH).

**Methods:**

This is a retrospective study among children between 2 months and 14 years of age discharged from Gondar University Hospital. All patient records discharged with a diagnosis of meningitis at GUH were reviewed from September 2011 - September 2013. The data was collected using a structured questionnaire from the patient record charts and analysis was done using SPSS-20.

**Results:**

80 cases (1.6%) of CABM out of 4996 admissions were identified. There were 60 (75%) cases of CABM using WHO criteria of cerebrospinal fluid leukocytosis (CSF) > 100cells/mm^3^, or 10-100cells/mm^3^ with either hypoglycorrhea or increased protein; and 20 (25%) with culture confirmation. *S. Pneumoniae* was the most frequent pathogen identified in 14 (70%) children. The most common age group were infants 2-12 month old (n = 32, 40%). Children with adverse outcomes had shown a higher frequency of being older children (p = 0.045), loss of consciousness (p = 0.046), seizure at admission (p < 0.01), and a positive CSF culture (p = 0.03).

**Conclusion:**

Introduction of PCV-10 has shown a decreased admission rate, mortality, and neurologic sequelae due to CABM.

## Introduction

Community acquired bacterial meningitis is a life threatening infection of the leptomeninges often related with serious complication and sequelae. Low and middle-income countries account for 98% of the estimated 5.6 million disability-adjusted life years attributed to meningitis globally. In high-income countries, bacterial meningitis ranks among the top ten causes of death in children younger than 14 years of age [1]. Primary prevention of meningitis using vaccines is paramount, since death and long-term disabling sequelae are substantial in all settings especially those with least access to health care [2]. Bacterial meningitis accounts for approximately 6-8% of hospital admissions in Ethiopia with a case fatality rate of as high as 22-28 [3]. The most common pathogens being *S. pneumonia, N. meningitides* and *H.influenzea* [4-6]. During the last several decades disease epidemiology and clinical features has changed dramatically in the countries that adapted the conjugate vaccines against *H. influenza type b* and *S. pneumoniae* [7-11]. In Ethiopia the implementation of Hib and PCV-10 started in 2007 and 2011, respectively. However, the impact of these vaccines on the disease epidemiology and clinical features are investigated in few international studies. Knowledge about the clinical and epidemiologic features will help practicing physicians to suspect and diagnose CABM as early as possible. Knowledge about factors associated with poor prognosis are valuable in selecting patients for more intensive monitoring and treatment. Especially in resource constrained settings where such equipment may be limited. The aim of this study was to assess the epidemiological and clinical features of CABM in our environment and to evaluate the short term outcome of CABM in Gondar University Hospital after the introduction of PCV-10 and Hib in our country.

## Methods

This retrospective study was conducted at Gondar University Hospital (GUH). Gondar is a city located in the North West Ethiopia, which is in the sub-Saharan meningitis belt. Most of the patients come from Gondar, but also receives patients from the adjoining regions of Amhara Regional State. The Gondar University Hospital is the largest hospital in Gondar. It is a 400-bed teaching hospital associated with a medical college. The department of pediatrics and child health has 5 different wards the main pediatric main ward has 46 beds and provides inpatient care for children < 14 years of age. All admissions and discharges to the pediatric main ward were reviewed over a 2 years period from September, 2011 through September, 2013. Potential cases were identified from the admission registration books after which the clinical and laboratory data were obtained from the individual case records. The cases were chosen based on the inclusion criteria of those patients older than 2 month and younger than 15 years and who fulfill the case definition of acute bacterial meningitis and have a full documentation. After the cases were chosen the following data was obtained from the records of the individual cases: 1) relevant clinical parameters including month of admission, demographic data, clinical features, risk factors, duration of symptoms, preadmission antibiotics and duration of stay; 2) treatment characteristics like choice antibiotics, dexamethasone and duration of treatment; 3) microbiological data: Gram stain, culture, cellular profile as well as relevant biochemical data from the CSF; 4) outcome variables comprising of death, short term complications and disappearance

### Operational definitions

**Case definition:** Both probable and confirmed bacterial meningitis cases according to World Health Organization definition were included [12].

**Probable meningitis:** Defined as culture-negative results but with the typical finding in cerebrospinal fluid: leukocytosis (polymorphonuclear leucocytes) > 100 cells/mm^3^ leukocytosis (polymorphonuclear leucocytes) 10-100cells/mm^3^and either elevated protein (> 100mg/dL) or decreased glucose (< 40mg/dL).

**Confirmed meningitis:** Laboratory-confirmed by growing (culture) or identifying (by Gram stain or antigen detection methods) a bacterial pathogen in the CSF or from the blood in a child with clinical syndrome consistent with bacterial meningitis

**Poor short term outcome:** Is defined as death or an adverse neurological outcome that was noted at the time of discharge from the hospital and that was attributable to the episode of meningitis

**Data analysis:** The clinical and epidemiologic data were analyzed using SPSS-20. The Chi square or Fisher exact test were used were used for assessing putative associations with adverse outcome. The confidence intervals relative risk and odds ratio were obtained as applicable. Significant association were presumed if P < 0.05. Binary logistic regression was used to identify the risk variables for the development of complications or death. For all the tests we considered a minimum significance level of 5%, with a power of 95%. The study was approved by the Institutional Review Board at Gondar University Hospital.

## Results

Over the past two years 80 children were discharged with the diagnosis of CABM. During this time there were 4994 admissions, giving an admission rate for CABM 1.6% during the study period. A peak admission rate was registered during the month of March (n=18, 22.5%) followed by February (n = 12, 15%) coinciding with the dry season. Bacterial meningitis was more common in children under one year (40%), and most patients were male (65%). Distribution of residence was slightly higher among urban and rural areas (53% vs 47%). Other demographic and clinical characteristics are shown in Table 1. Possible predisposing factors for meningitis were upper respiratory traction infection (25%), lack of breastfeeding (10%), previous admission (10%), lack of immunization (10%) and malnutrition of both moderate and severe in 2.5%. Of the children who werenot vaccinated or partially vaccinated, 4(50%) of them were infants and the rest are above one year of age. Only 12 (15%) of patients were admitted to our hospital within 24 hour of the onset of symptoms, the majority presented after 24 hours but within 72 hours (n = 34, 42.5%) or after 72 hours (n = 34, 42.5%). Among those admitted 22 (27.5%) received some form of antibiotics in local health centers The most common clinical manifestations for each age group are shown in Table 2. Almost all patients (n = 78, 97.5%) had CSF examination done immediately after admission, the results are shown in Table 2. Moderate pleocytosis (>1000cells/mm^3^) was found in 20% of children, the majority of the patients (75%) had cell count between 100-1000, cells/mm^3^ with a neutrophil predominance (70-98%). Hypoglycorrhea and elevated protein level was fond in 42.5%. Of 80 patients diagnosed with CABM, 20 (25%) of cases had culture confirmed ABM, *S. pneumoniae* was isolated in 14/20 (70%) and *H. influenzea* in 4/20 (20%) of the cases.

**Table 1:** Baseline demographics of 80 children admitted with community-acquired bacterial meningitis to Gondar University Hospital

Patient characteristic	Overall n (%)
**Age group**	
2-12 months	32 (40)
1-5 years	16 (20)
6-10 years	16 (20)
10-15 years	16 (20)
**Sex**	
Male	52 (65)
**Residence**	
Urban	43 (53.7)
Rural	37 (46.3)
**Immunization status**	
Fully immunized	72 (90)
Partially immunized	4 (5)
No immunizations	4 (5)
**Feeding practice children < 1 year**	
Breastfed	72 (90)
No breastfeeding	8 (10)
**Duration of illness before presentation**	
<24 hours	12 (15)
24-72 hours	34 (42.5)
> 72 hours	34 (42.5)
**Clinical features**	
Fever,	80 (100)
Vomiting	78 (97.2)
Headache	30 (37.5)
Neck stiffness	36 (45)
Seizure	32 (40)
Loss of consciousness	26 (32.5)
Bulged fontanel	
Kerning sign	14 (17.5)
14 (17.5)	
Brudzinski sign	14 (17.5)
**Prior antibiotic use**	
Yes	22 (27.5)
No	58 (72.5)
**Antibiotic choice**	
Ceftriaxone	50 (62.5)
Penicillin + chloramphenicol	30 (37.5)

**Table 2:** Age group distribution of symptoms of community-acquired bacterial meningitis at onset

Clinical manifestation	Age group 2mo-12 mo n (%)	Age group 1-5yrs n (%)	Age group 6-10 yrs n (%)	Age group >10 yrs n (%)
Fever	32 (100)	16 (100)	16 (100)	16 (100)
Coma	6 (18.75)	6 (37.5)	6 (37.5)	8 (50)
Seizure	8 (25)	12 (75)	8 (50)	4 (25)
Neck stiffness	4 (12.5)	12 (75)	10 (62.5)	10 (62.5)
Headache	0	4 (25)	10 (62.5)	16 (100)
Vomiting	32 (100)	16 (100)	14 (87.5)	16 (100)

Age related clinical and laboratory features of bacterial meningitis are shown in Table 2 and Table 3. There is no specific sign or symptom which can predict culture positivity as it's shown on analysis of symptoms versus the culture positivity rate Table 4. The majority of patients (n = 50, 62.5%) were treated with ceftriaxone and the rest with a combination of penicillin and chloramphenicol (n = 30, 37.5%). Just more than half of the patients (52.5%) were treated for 10 days and 28(35%) of them were treated for 14 days. Dexamethasone was given for 56(70%) patients before the antibiotics or at the same time. Most patients (n=68, 85%) were discharged improved and the rest (n = 12, 15%) were discharged with sequelae, died or disappeared. Overall case fatality was 7.5% (6/80) and all the deaths were due to *S.pneumoniae* which gives a case fatality rate for this organism of 42.8%. All of the children who died were above the age of 10 years (P < 0.05). Prolonged fever is found to be more prevalent among children under five years followed by infants and older than 10 years (p = 0.36). Seizure after 72 hrs of admission was a common complication among children older than 10 years old (p = 0.283).

**Table 3:** Age group distribution of cerebral spinal fluid characteristics in children with community-acquired bacterial meningitis

CSF parameter	Age group2mo-12 mo n (%)	Age group1-5yrs n (%)	Age group6-10 yrs n (%)	Age group>10 yrs n (%)
**Cell count**	32 (100)	16 (100)	16 (100)	16 (100)
**Gram stain**	10 (31.25)	6 (37.5)	0	6 (37.5)
**Culture**	8 (25)	6 (37.5)	0	6 (37.5)
**Low glucose**	10 (31.25)	6 (37.5)	6 (37.5)	12 (75)
**High protein**	10 (31.25)	6 (37.5)	6 (37.5)	12 (75)

**Table 4:** Clinical parameters according to cerebral spinal fluid isolates in children with community-acquired bacterial meningitis

Manifestation	Culture negative (n=60)	S. pneumoniae (n=14)	H. influenzea (n=4)	Other (n=2)^1^	P-value
Fever	60 (100%)	14 (100%)	4 (100%)	2 (100%)	-
Objective fever	40 (66.7%)	14 (100%)	4 (100%)	2 (100%)	0.217
Vomiting	58 (96.67%)	14 (100%)	4 (100%)	2 (100%)	0.952
Headache	22 (36.67%)	6 (42.9%)	2 (50%)	0	0.843
Neck stiffness	28 (46.67%)	4 (28.6%)	4 (100%)	0	0.253
Seizure	18 (30%)	10 (71.4%)	4 (100%)	0	0.05
Coma	12 (20%)	12 (85.7%)	2 (50%)	0	0.08
Abnormal cry	28 (46.67%)	2 (14.3%)	0	0	0.609
Failure to suck	12 (20%)	4 (28.6%)	2 (50%)	2 (100%)	0.249
Bulged fontanel	12 (20%)	2 (14.3%)	0	0	0.846
Focal signs	0	2 (14.3%)	0	0	0.184

1 other: group A streptococcus

Different factors contributed for poor outcome in this study as it is shown on Table 5. Neurologic sequelae (focal neurologic deficit, depressed consciousness) at discharge was associated with young age < 5 years (OR = 1.2, 95% CI 0.05-1.3, p = 0.045), culture positivity (OR = 1.3, 95%CI 1.2-3, p = 0.004) and focal neurologic deficit at admission (OR = 5 95% CI 2.1-5.2, p < 0.001).Children presenting with loss of consciousness had a risk of seizure after 72 hr (OR = 7.8, 95% CI, 1.7-8.7, P < 0.001). Seizures at admission was found to be strongly associated with the development of seizure after 72 hr (OR = 12, 95% CI 1.07 - 13, p=0.04), and prolonged fever (OR = 1.2, 95% CI 0.09-2.14, p = 0.01).Culture positivity was found to have an association relation with prolonged fever (OR = 1.3, 95% CI, 0.1-1.8 p = 0.01), prolonged seizure (OR=2.1 95% CI, 1.8-4.9, p = 0.01) and death (OR = 2.4, 95% CI 0.27-3.8, p < 0.01). Choice of antibiotics was found to have a strong correlation with death. Use of penicillin with chloramphenicol was found to have a high risk of death (OR = 4.1, 95%CI, 2.5-5.4, p = 0.04).

**Table 5:** Analysis of prognostic factors for poor short-term outcome of 80 patients with community acquired bacterial meningitis

Prognostic variables	Sequelae (n=80)	Adjusted OR95% CI	P-value
Loss of consciousness	14 (17.5%)	7.8 (0.723-1.702)	0.046
Seizure at admission	32 (40%)	12 (1.005-1.769	<0.010
Culture positivity	20 (25%)	1.3 (0.286-0.860)	0.030
Antibiotic choice	4 (5%)	4.1 (0.527-0.546)	0.042
Age	4 (5%)	12 (0.053-0.197)	0.045

## Discussion

The hot, dry seasonal increase in admission and male predominance was consistent with other studies from Bangladesh [13] and BurkinaFaso [14]; but not similar to previous studies from Gondar [4] and Addis Abeba [3]. The preponderance of the under-fives underscores the age group that bears the brunt of the disease burden. Clinical features that are found to be dominant in our study are also found in the previous studies done in the pre PVC period suggesting that there is no significant change in the clinical presentation of CABM after the introduction of the vaccine [15]. We used a retrospective analysis of clinical and epidemiological features to document a reduction in admission rate of CABM by 62.7% (from 4.3% to 1.6%) and case fatality rate of 56.1% (from 17.1% to 7.5%) when compared to previous studies which were done prior to the introduction of PCV-10 [16]. Some of the reduction in death rate could still be due to the improvement in the general pediatric care. Consistent with researches done in the pre PVC-10 era in Tikur Ambessa, Gondar and Awassa University Hospitals, *S. pneumoniae* is still the most common organism isolated from the culture accounting for the 70% [4, 17]. Part of this may be explained by the fact most cases of pneumococcal meningitis was seen in children older than 10 years, and those who were partially immunized or unimmunized. Finally, other strains not contained by the vaccine could be the pathogen. Literature in the pre PVC-10 period indicates that CABM is associated with a high risk of neurological complications, with a percentage of up to 38%, and mortality rates up to 18% [17-20]. In the present study, this frequency was 5% for the complication and mortality rate was 7.5%, which is significantly lower than seen in previous studies.

In previous studies loss of consciousness, focal neurologic deficit, seizure at admission, delayed presentation and *S. pneumoniae* were found to be risk factors for poor outcome [19-23]. In the present study, the presence of seizure at admission, loss of consciousness, culture positivity and focal neurologic deficit were associated with poor outcome which was in agreement to these previous studies. However, use of dexamethasone and delayed presentation was not found to be a predictor of poor outcome in this study. Antibiotics of choice were found to have a strong association with death in the present study. This may reflect an increase in possible resistant strains in the community but it needs a laboratory baser surveillance study for to make conclusion. While third generation cephalosporins such as ceftriaxone are the first line treatment for CABM in our setting, it may not be available due to stock outs, resulting in health care providers using older treatment recommendations from WHO such as penicillin and chloramphenicol. The study was retrospective in design and subject to the potential biases of retrospective reviews. However, we collected objective clinical and laboratory features that should be reliably recorded in the medical record. The medical records also had limited data about the study patient's PCV-10 immunization status. Although overall immunization was high in the study we cannot exclude the possibility that some of our study patients were either unimmunized or under immunized with PCV-10. The other limitation was the subjectivity of discharge conditions has created some difficulties in finding the exact outcome, especially of neurologic outcomes. Another limitation was the paucity of data regarding antibiotics susceptibility patterns, since laboratory reports were incomplete. Finally, this study had only 20% culture positive specimens, though this would result in an overestimation of CABM. The limited availability of culture reflects many resource-constrained setting, and we believe by applying WHO criteria for the definition of CABM gives us a reasonable estimate of the burden of CABM in our community.

## Conclusion

Based on the results of this study, we can conclude that introduction of PCV-10 and Hib to the immunization schedule has decreased the admission rate, mortality, and morbidity of CABM among children under 5 years old. It is also possible to conclude from this study that loss of consciousness, seizure at admission and focal neurologic deficit at admission predictors of adverse outcomes at admission and hence these patients needs proper evaluations and meticulous follow up in the ward as well as after discharge.

### What is known about this topic

- The etiology and clinical features of bacterial meningitis;
- Possible outcome of patients with bacterial meningitis.

### What this study adds

- The change in the epidemiology after the introduction of pneumococcal vaccine;
- The change in clinical feature and outcome of bacterial meningitis after pneumococcal vaccine.

## Competing interests

The authors declare no competing interests.
